# Differential Effects of CSF-1R D802V and KIT D816V Homologous Mutations on Receptor Tertiary Structure and Allosteric Communication

**DOI:** 10.1371/journal.pone.0097519

**Published:** 2014-05-14

**Authors:** Priscila Da Silva Figueiredo Celestino Gomes, Nicolas Panel, Elodie Laine, Pedro Geraldo Pascutti, Eric Solary, Luba Tchertanov

**Affiliations:** 1 Laboratoire de Biologie et de Pharmacologie Appliquée, École Normale Supérieure de Cachan, Cachan, France; 2 Instituto de Biofísica Carlos Chagas Filho, Universidade Federal do Rio de Janeiro, Rio de Janeiro, Rio de Janeiro, Brazil; 3 Institut Gustave Roussy, Villejuif, France; 4 Faculty of Medicine, Paris- Sud University, Le Kremlin-Bicêtre, France; 5 Centre de Mathématiques et de Leurs Applications, École Normale Supérieure de Cachan, Cachan, France; Oak Ridge National Laboratory, United States of America

## Abstract

The colony stimulating factor-1 receptor (CSF-1R) and the stem cell factor receptor KIT, type III receptor tyrosine kinases (RTKs), are important mediators of signal transduction. The normal functions of these receptors can be compromised by gain-of-function mutations associated with different physiopatological impacts. Whereas KIT D816V/H mutation is a well-characterized oncogenic event and principal cause of systemic mastocytosis, the homologous CSF-1R D802V has not been identified in human cancers. The KIT D816V oncogenic mutation triggers resistance to the RTK inhibitor Imatinib used as first line treatment against chronic myeloid leukemia and gastrointestinal tumors. CSF-1R is also sensitive to Imatinib and this sensitivity is altered by mutation D802V. Previous *in silico* characterization of the D816V mutation in KIT evidenced that the mutation caused a structure reorganization of the juxtamembrane region (JMR) and facilitated its departure from the kinase domain (KD). In this study, we showed that the equivalent CSF-1R D802V mutation does not promote such structural effects on the JMR despite of a reduction on some key H-bonds interactions controlling the JMR binding to the KD. In addition, this mutation disrupts the allosteric communication between two essential regulatory fragments of the receptors, the JMR and the A-loop. Nevertheless, the mutation-induced shift towards an active conformation observed in KIT D816V is not observed in CSF-1R D802V. The distinct impact of equivalent mutation in two homologous RTKs could be associated with the sequence difference between both receptors in the native form, particularly in the JMR region. A local mutation-induced perturbation on the A-loop structure observed in both receptors indicates the stabilization of an inactive non-inhibited form, which Imatinib cannot bind.

## Introduction

Receptor tyrosine kinases (RTKs) are cell-surface transmembrane receptors that possess a tightly regulated tyrosine kinase (TK) activity within their cytoplasmic domain [1]. They act as sensors for extracellular ligands, the binding of which triggers receptor dimerization and activation of the kinase function, leading to the recruitment, phosphorylation and activation of multiple downstream signaling proteins, which ultimately govern the physiology of cells [2]. Based on their overall architecture and kinase domain (KD) sequence, RTKs have been grouped into 20 subfamilies [3]. The type III RTK subfamily includes the stem cell factor (SCF) receptor KIT, the macrophage colony-stimulating factor-1 (CSF-1) receptor CSF-1R (or FMS), the platelet-derived growth factor α and β (PDGFR-α and PDGFR-β) and the FMS-like tyrosine kinase 3 (FLT3) [3], [4]. As compared to *KIT*, whose activating mutations are hallmarks of systemic mastocytosis [5], and gastro-intestinal stromal tumors (GISTs) [6], or to *FLT3*, whose activating mutations are frequently observed in acute myeloid leukemias (AML) [7], activating mutations in *CSF-1R* gene have been rarely detected in human tumors [8]. Nevertheless, CSF-1R is a therapeutic target in oncology, either to inhibit a paracrine loop that promotes tumor growth [9] or to re-educate tumor associated macrophages (TAMs) within tumor microenvironment [10]. The receptor could be targeted also to prevent osteopenia in neurofibromatosis type-1 [11], whereas the diverse constitutive heterozygous mutations in CSF-1R that are responsible for an autosomal dominant neurological disorder called HDLS (hereditary diffuse leukoencephalopathy with spheroids) are loss of function mutations [12].

**Figure 1:**
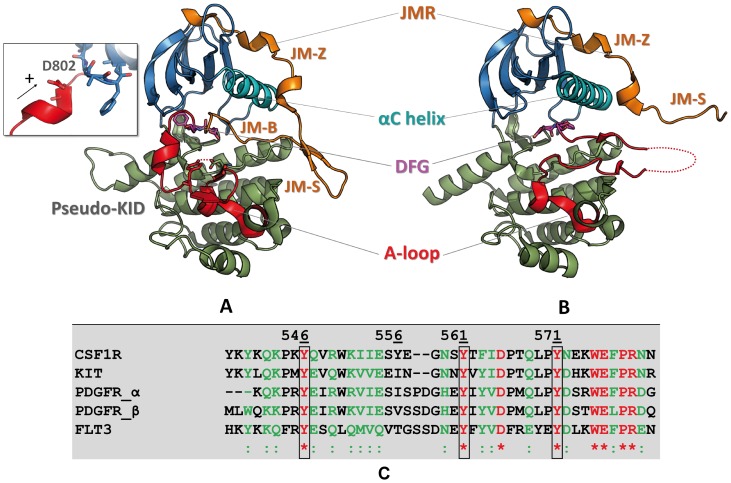
Top. Structure of CSF-1R cytoplasmic region. Crystallographic structures of the native receptor (A) in the inactive (2OGV[22]) and (B) the active forms (3LCD [85] are presented as cartoon. The different domains of CSF-1R and key structural fragments are highlighted in color. The N-terminal proximal lobe (N-lobe) is in blue, the C-terminal distal lobe (C-lobe) is in green, the Cα-helix is in cyan, the activation loop (A-loop) is in red, the juxtamembrane region (JMR) is in orange. The DFG motif (Asp796, Phe797, Gly798) and position of D802V mutation (insert) are represented in sticks. Bottom. The JMR sequence in RTKs of III family. The sequence alignment shows a poor sequence conversation of the JMR among the receptors TK of type III. Identical residues and similar residues are shown in red and in green, respectively. The three strongly conserved tyrosine residues are contoured. Except CSF-1R, the other RTKs from III family possess a second functional phosphotyrosine (green, contoured) in JM-Switch.

The type III RTKs have a common architecture that includes extracellular immunoglobin (Ig)-like domains to which polypeptide ligands bind, a single-pass transmembrane helix, an autoinhibitory juxtamembrane region (JMR) and a cytoplasmic tyrosine kinase (TK) domain including a kinase insert domain (KID) [13], [14] of a variable length (∼60–100 residues), and a carboxy-terminal tail [15]–[17] (**Fig. S1**). The TK domain has a bi-lobar structure, with an ATP-binding cleft located between the N- and C-terminal lobes. The N-lobe is composed of twisted five-stranded anti-parallel β-sheet adjacent to an α-helix (Cα-helix) and the C-lobe shows predominantly α-helical structure (**Fig. 1**). The C-lobe contains an activation loop (A-loop) that begins with the highly conserved ‘DFG’ motif composed of three amino acids − aspartic acid (D), phenylalanine (F), and glycine (G).

In the absence of ligand, the receptors are in dynamic equilibrium between two states: the inactive autoinhibited state that is highly dominant, and the active state [18], [19]. Two crucial kinase regulatory segments, the A-loop and the JMR, undergo extensive conformational rearrangements during the activation/deactivation processes (**Fig. 1**). In the inactive auto-inhibited state of the receptor, the A-loop is adjacent to the active site and the DFG motif at its N-extremity adopts an “out” conformation, *i.e.*, its phenylalanine is flipped into the ATP-binding site, thus preventing ATP and Mg^2+^ co-factor binding [20], [21]. This conformation is stabilized by the JMR that inserts itself directly into the kinase active site and impairs the arrangement of the A-loop in its active conformation. The single tyrosine in the A-loop binds to the catalytic loop as a pseudo-substrate and contributes to keep the receptor in its inactive form. Upon activation, the JMR moves from its auto-inhibitory position to a completely solvent-exposed emplacement. This is followed by a conformational swap of the A-loop from its inactive packed arrangement to an active extended conformation. Such large-scale conformational transition, together with a switch of the DFG motif to an “in” conformation allows ATP entrance and binding in the catalytic site.

Analysis of the crystallographic structures of KIT, CSF-1R and FLT3 in their inactive state [20]–[22] suggested a particular mechanism of auto-inhibition based on extensive interactions of the JMR with the TK domain. The JMR is composed of three fragments: JM-Binder (JM-B), buried into the TK domain making direct contacts with the Cα-helix, the catalytic (C-) loop and the A-loop; JM-Switch (JM-S) that adopts a hairpin-like conformation positioned apart from the C-lobe and contains the tyrosine residues responsible for the conformational switch; and JM-Zipper (JM-Z), packed along the solvent-exposed face of the Cα-helix (**Fig. 1**). Together, the JM-B and the JM-Z block the Cα-helix, which also regulates the catalytic activity of the kinases [23], and prevent the A-loop from adopting an active conformation, restricting the inter-lobe plasticity.

Mutational hotspot regions in type III RTKs are mainly located in the JMR and the A-loop, although mutations have also been found in the extracellular and in the transmembrane regions [17]. Gain-of-function point mutations induce either tyrosine kinase constitutive (*i. e.*, ligand-independent) activation and/or resistance to the tyrosine kinase inhibitors. Particularly, mutation of an aspartic residue in the A-loop, namely D835(V/Y/H/V/E/N) in FLT3, D816(V/H/N/Y/E/I) in KIT, and D842V in PDGFRα, is a typical example of mutation that confers a proliferative signal. The KIT D816V oncogenic mutation in addition triggers resistance to the RTK inhibitor Imatinib [6], [24], [25]. CSF-1R is also sensitive to Imatinib and this sensitivity is altered by D802V mutation [26]. These mutations reverse the conformational equilibrium of the kinase toward the active form, which compromises the efficacy of the inhibitors targeting inactivated form of the receptor [27]. The physiological role of D802V mutation is not well-understood, the previous studies have shown that D802V mutation constitutively activates the receptor, transforming the haemopoietic cell line FDC-P1 yet prevented Rat-2 fibroblast transformation, apparently due to a higher rate of receptor degradation [28], [29]. Considering the differential physiological effects induced by the equivalent mutation in the two receptors, a dissimilar role of the equivalent residue, D802 in CSF-1R and D816 in KIT, in the activation mechanisms may be suggested.

CSF-1R and KIT have considerable sequence identity (68%) and their auto-inhibited states display great structural similarities (RMSD is 1.14 Å) [22]. Unlike the other type III RTK family members, the JM-S region of CSF-1R contains a unique conserved tyrosine (Y561) [30] (**Fig. 1**), which is responsible for the receptor activation.

We have reported that KIT D816V mutation, positioned in the A-loop, induced a long-range structural reorganization of the JMR, followed by its release from the KD in the absence of extracellular ligand binding [31]. We have also evidenced that a communication route established between the distant A-loop and JMR in the native protein was disrupted in KIT D816V mutant [32]. In the present paper, we explore the impact of the equivalent mutation D802V on the structure, dynamics and stability of CSF-1R by all-atom molecular dynamics (MD) simulations, principal component analysis (PCA), normal modes analysis (NMA), binding free energy evaluation and inter-residue communication analysis. The data produced for CSF-1R were carefully compared with those previously obtained for KIT. Although D802V mutation in CSF-1R seems to have a more subtle impact on the receptor structure respectively to KIT, it induces a loss of allosteric communication of the JM-B with the main regulatory fragments − the A-loop and the Cα-helix − similarly to KIT. Nevertheless, a part of the JMR communication with the KD observed in the native receptor is preserved in CSF-1R mutant, and probably participates in controlling CSF-1R activation. The mutation-induced disruption of a small 3_10_-helix in the A-loop and reorganization of the DFG motif conformation is consistent with the inactive non-autoinhibited conformation observed in both receptors. The results of this study offer a plausible common mechanism, according to which the equivalent mutation may induce resistance to the tyrosine kinase inhibitors targeting the inactive autoinhibited state of type III RTKs.

## Materials and Methods

### Bioinformatics and structure analysis

#### Secondary structure prediction

The secondary structure prediction for the JMR residues was performed using six methods based on the protein primary sequence:

- GOR4 [33], an information theory-based method that uses probability parameters derived from empirical studies of crystallographic structures, taking into account not only the propensities of individual amino acids to form particular secondary structures, but also the conditional probability given that its immediate neighbors have already formed that structure.
- Jpred [34] uses the Jnet [35] algorithm to make the prediction of the secondary structure and solvent accessibility by combining BLAST [36], to search the protein sequence against sequences in the Protein Data Bank (PDB) [37] and Uniref90 [38] − in this case, only Uniref90 database was used; PSI-BLAST [39], to make an alignment; HMMer [40], to construct an hidden Markov model profile based on the alignment; and a Position-specific scoring matrix (PSSM) [41], output from PSI-BLAST.
- SOPMA [42] makes the prediction of the secondary structure based on the homolog method of Levin [43]. The algorithm uses a database of 126 chains of non-homologous proteins to search against the user sequence.
- SCRATCH [44] combines machine learning methods, evolutionary information in the form of profiles, fragment libraries extracted from the PDB and energy functions to predict protein structural features and also tertiary structures, see the article for further information of the specific methods used by each predictor.
- NetSurfP [45] consists of two neural network ensembles used to predict the secondary structure and the relative surface accessibility of an amino acid.
- Psipred [46] incorporates two feed-forward neural networks which perform an analysis on output obtained from PSI-BLAST.

Finally, STRIDE, a knowledge-based algorithm that assigns the secondary structure from atomic coordinates based on the combined use of hydrogen bond energy and statistically derived backbone torsional angle information [47] was used to predict the secondary structure of JMR in CSF-1R using the crystallographic structure (2OGV) [22] as input.

VMD [48] and PYMOL [49] were used for visualization and the analysis graphics were drawn using Grace (http://plasma-gate.weizmann.ac.il/Grace/).

#### Electrostatic potential surface

Electrostatic potential surfaces were calculated on the crystal structures of CSF-1R (PDB code: 20GV) and KIT (PDB code: 1T45) using APBS at PDB2PQR web-based server (http://www.poissonboltzmann.org/pdb2pqr/d/web-servers).

### Molecular dynamics simulations

**Preparation of initial coordinate files.** The crystallographic structure of the wild-type (WT) auto-inhibited form of CSF-1R cytoplasmic region (PDB id: 2OGV) [22] was retrieved from the Protein Data Bank [37]. All crystallographic water molecules were removed. MODELLER 9v8 [50], [51] was used to add missing atoms at some residues (543–545, 606–607, 620–621, 623, 625, 677, 741, 812, 814 and 918). *In silico* substitution of Asp (D) to Val (V) at position 802 was performed by MODELLER, using the WT structure as template, making them comparable starting models. Generated models of the native CSF-1R and its mutant D802V were referred to as CSF-1R^WT^ and CSF-1R^MU^ respectively.

**Set up of the systems.** The setup of the systems (CSF-1R^WT^ and CSF-1R^MU^) was performed using AMBER force field, parameter set 99SB [52] inside GROMACS package, version 4.5 [53]. The molecules were centered in a cubic box with a 1.5 nm distance to the faces, under periodic boundary conditions and solvated with explicit TIP3P model water molecules [54]. Cl^−^ counter ions were added when necessary to neutralize the overall charge (3 for CSF-1R^WT^ and 4 for CSF-1R^MU^). The minimization procedure consisted of 2 steps: steepest descent energy minimization (EM) with the solute atoms restrained; (ii) EM with all atoms free. The equilibration procedure was performed on the solvent, keeping the solute heavy atoms restrained for 500 ps at 310 K and a constant volume (canonical NVT ensemble).

**Production of trajectories.** Two production runs of 50 ns were carried out for both receptors, CSF-1R^WT^ and CSF-1R^MU^. The temperatures of solute (protein) and solvent (water and ions) were separately coupled to the velocity rescale thermostat [55] at 310 K with relaxation time of 0.1 ps. The pressure was maintained at 1 atm by isotropic coordinate scaling with relaxation time of 1 ps using Berendsen thermostat [56]. A time step of 2 fs was used to integrate the equations of motion based on the Leap-Frog algorithm [57]. The Lennard-Jones interactions were shifted to a cut-off 1.4 nm, and the Particle Mesh Ewald (PME) method [58] was used to treat long-range electrostatic interactions. The neighbor list for the electrostatic interactions was updated every 5 steps, together with the pair list. All bonds were constrained using the P-LINCS algorithm [59]. The SETTLE algorithm [60] was used to constrain the geometry of the water molecules. Coordinates files were recorded every 1 ps.

**Analysis of the trajectories.** The trajectories for each pair of molecular dynamics (MD) simulations were analyzed with tools included in the GROMACS package. When concatenating the MD simulations replicas, the first 5 ns of each replica trajectory needed to achieve relaxation were not considered. Analyses were performed on the resulting merged trajectory of 90 ns for each protein or based on the 45 ns individual replicas. We have also produced a 60 ns concatenated trajectory from the last 30 ns from each replica to be further used for *IDSs* calculations with MONETA [32]. A convergence analysis was performed on the merged trajectories using an ensemble-based approach [61]. The algorithm makes use of the global Cα atoms RMSD to discriminate representative MD conformations. The procedure for each trajectory can be described as follows: (i) a set of *reference* structures are identified, (ii) the MD conformational ensemble was clustered into corresponding *reference* groups. Each *reference* structure was first picked up at random and associated with a bin of conformations distant by less than an arbitrary cutoff *r*. Then the merged trajectory was split in four halves (two halves for each replica) and conformations from each half were grouped based on their RMSD from each reference structure. A good convergence quality was assessed when each reference group was populated by conformations from the four halves of the trajectory at equivalent levels, meaning that every reference structure is equivalently represented in both replicas of the trajectory.

**Geometrical measurements.** Two characteristic distances were monitored every 10 ps over the MD simulations of each model: (i) the distance d1 between the centroid (C) of the JM-B region (residues 543–552, C1) and the C of the remaining residues in the N-lobe (582–664, C1′); (ii) the distance d2 between the C of the JM-S (residues 553–564, C2) and the C-lobe (residues 671–922, C2′). The hydrogen (H-) bond analyses were done with the program g_hbond available in GROMACS. Time occupancy of H-bonds stabilizing the JMR and the A-loop was recorded every 100 ps of simulation for each model of CSF-1R. H-bonds (D•••H–A) where defined with a DHA angle cutoff of 120° and a D•••A distance cutoff of 3.5 Å (D and A are donor and acceptor atoms).

**Secondary structure prevalence.** The secondary structure profile was calculated using the program do_dssp available in GROMACS. The program makes use of DSSP [62]. The calculation was performed over the merged 90 ns trajectories for both forms of the receptor.

### Energy analysis

The free energy of JMR or its segments (ligand, L) binding to KD (receptor, R) defined as
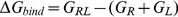
(1)was computed over the merged trajectories and on the individual MD simulations, considering only the last 30 ns from each replica for both CSF-1R^WT^ and CSF-1R^MU^. Free energies were evaluated using the Molecular Mechanism Generalized Born Surface Area (MMGBSA) method, implemented in AMBER 12 [63]–[66]. This method combines the molecular mechanical energies with the continuum solvent approaches. The molecular mechanical energies represent the internal energy (covalent bonds, angles and dihedral angels contributions), and contribution of van der Waals and electrostatic interactions. The electrostatic contribution to the solvation free energy is calculated by generalized Born (GB) methods. The non-polar contribution to the solvation free energy is determined with solvent-accessible-surface-area-dependent terms. Estimates of conformational entropies are calculated with the normal mode module from AMBER.

### Normal modes analysis

Normal modes (NM) analysis was performed using the diagonalization in a mixed basis (DIMB) method [67] of the VIBRAN module of CHARMM 35b3 [68], [69] on MD conformations from (i) CSF-1R^WT^ taken at 1 526, 49 390, 66 530 and 81 680 ps, spanning both replicas contained in the 90-ns merged trajectory, and (ii) CSF-1R^MU^ mutant taken at 5 510, 23 530, 40 670 and 84 680 ps. The selected MD conformations were found to be the most representative of the trajectories, according to the convergence analysis. The first hydration shell (5 Å) around the MD conformations was kept to help prevent the solvent-exposed regions of the protein from collapsing during the minimization procedure [70]. During initial steepest descent energy minimization of the system, mass-weighted harmonic constraints of 250 kcal/mol/A^2^ were applied to the starting structure and reduced by a factor of 2 every 1000 minimization steps until they fell below a threshold value of 5 kcal/mol/A^2^. The constraints were then removed and the system was minimized by conjugate gradient and adopted-basis Newton-Raphson steps until the RMS energy gradient fell below 10^−5^ kcal/mol/A^2^. Normal modes were computed by diagonalizing the mass-weighted Hessian matrix of the energy-minimized conformations and the 96 non-zero lowest-frequency modes were analyzed. The degree of collectivity of the JMR motions in a given mode *l* was calculated as [71], [72]:
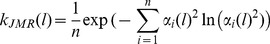
(2)where n = 663 is the number of atoms belonging to JMR. The quantity *α_i_* is defined as:
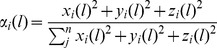
(3)where *x_i_*, *y_i_* and *z_i_* are the components of mode *l* showing the three degrees of freedom of atom *i* and such that 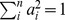. The degree of collectivity is comprised between 0 and 1. A value of *1/n* indicates that only one atom is involved in the motion while a value close to 1 indicates high collectivity.

The resultant displacement, *i.e.* the norm of the resultant displacement vector, of any fragment of the protein was calculated as:
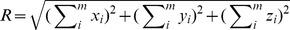
(4)over the ensemble *M* of the *m* atoms belonging to the fragment –172 for JM-Switch and 181 for JM-Zipper.

### Principal component analysis

A Principal Component Analysis (PCA) was applied to each model to identify the main eigenvectors (3N directions) along which the majority of the collective motions are defined. The calculations were performed on the backbone atoms positions recorded every ps along the trajectories for each 45 ns simulation replica. The 100 first modes of each trajectory were extracted. The calculation was performed using the g_covar module of GROMACS package. The overlap between the first 10 modes of each trajectory was calculated using the g_anaeig module of GROMACS package. Briefly, the method consists in overlapping the subspace spanned by ***m*** orthogonal vectors **w_1_**,…,**w_m_** with a reference subspace spanned by *n* orthonormal vectors **v_1_**, …,**v_n_** and it can be quantified as follows:
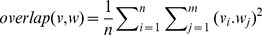
(5)


The overlap will increase with increasing *m* and will be 1 when set **v** is a subspace of set **w**.

### Analysis of intramolecular communication

Modular network representations of CSF-1R^WT^ and CSF-1R^MU^ were built and visualized with MONETA [32], using the most advanced version [73]. The principle of the MONETA approach consists in building a modular network representation of the protein, composed of clusters of residues representing *independent dynamic segments* (*IDSs*) and chains of residues representing *communication pathways* (*CPs*). The representation is derived from the protein topology and the inter-residue dynamical correlations calculated on a conformational ensemble obtained by MD simulations. *CPs* were generated based on the communication propensities [81] between all protein residues. *IDSs* and *CPs* in CSF-1R^WT^ and CSF-1R^MU^ were determined using a protocol described in details in our previous work [32]. *IDSs* were identified from Local Feature Analysis (LFA) [74] based on PCA. PCA calculations were performed for both models of the receptor, on the Cα atoms covariance matrices calculated on the concatenated 60 ns trajectory merged from the two 50 ns MD replicas, considering only the last 30 ns of each simulation. From the 3N eigenvalues associated with the 3N eigenvectors, the first 17 and 19 eigenvectors were sufficient to describe 80% of the total Cα atomic fluctuations on CSF-1R^WT^ and CSF-1R^MU^, respectively. These vectors were used to apply the LFA formalism as described in [32]. A threshold value P_cut_ was arbitrary chosen by the program to keep 1.0% of all LFA cross-correlations above it. The value was set to 0.035 for the WT and 0.038 for the D802V CSF-1R. Distance matrices consisting of the average of the smallest distance between each residue pairs were computed using the g_mdmat module of GROMACS package, v.4.5.6. Two residues were considered neighbors if the average smallest distance between them was lower than a given threshold d_cut_ of 3.6 Å. Since we have observed a slightly different dynamical behavior in the two MD simulation replicas, we have computed the *CPs* on the individual MD simulations, considering the last 30 ns only, in order to distinguish between the communication pathways of CSF-1R^WT^ and CSF-1R^MU^. One replica of each form of receptor was retained for the illustrations. The *CPs* definition was based on the concept of communication propensity described elsewhere [32]. The *CPs* are grown ensuring that the adjacent residues are connected by non-covalent interactions and that every residue in the *CP* is connected to any other point by a shorter commute time (*CT*). Non-bonded interactions were recorded along the MD simulations using LIGPLOT [75]. Two residues were considered as interacting when they formed at least one non-bonded interaction for 50% of the simulation time. To discriminate between large and short *CT*s, a threshold *CT_cut_* was chosen so that highly connected residues communicate efficiently with about 10% of the total number of residues in the protein [76]. The threshold values were set to 0.1 for both models.

Statistical analyses were performed with the R software [77]; visualization of the structure/interaction/communication characteristics/results are performed with PyMOL [49] incorporated in MONETA [73].

## Results and Discussion

Models of the native cytoplasmic region of CSF-1R (CSF-1R^543–922^) and its mutant D802V (referred to as CSF-1R^WT^ and CSF-1R^MU^ respectively) were generated from the crystallographic structure of the wild-type (WT) receptor in an auto-inhibited inactive state (2OGV) [22]. A similar KIT^WT^ and KIT^MU^ abbreviation was used for cross-receptor comparisons.

### Differential effects of CSF-1R D802V and KIT D816V homologous mutations on receptor tertiary structure

**Figure 2:**
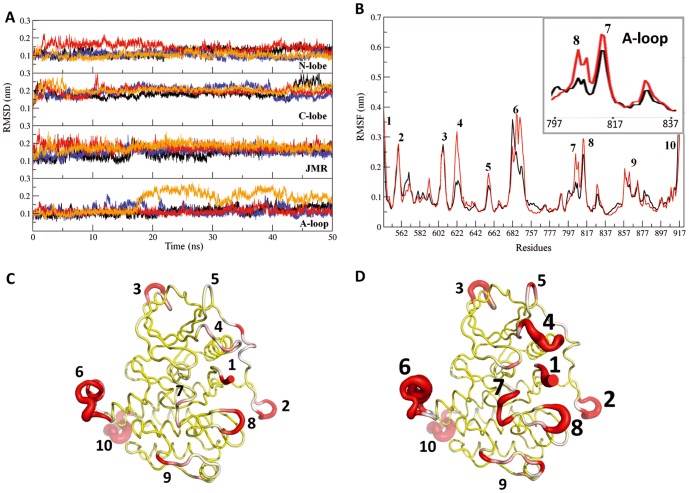
MD simulations of the CSF-1R cytoplasmic domain in the inactive state. Two forms of receptor, the native (CSF-1R^WT^ and CSF-1R^MU^ (D802V) were simulated twice during 50 ns. (**A**) The Root Mean Square Deviation (RMSD) values were calculated for backbone atoms from trajectories 1 and 2 of MD simulations of CSF-1R^WT^ (black and blue) and CSF-1R ^MU^ (red and orange). RMSDs (in *nm*) plotted versus simulation time (*ns*) and showed separately for N- and C-lobes, JMR and A-loop regions. (**B**) The Root Mean Square Fluctuations (RMSF) computed on the backbone atoms over the total production simulation time of CSF-1R^MU^ (red) were compared to those in CSF-1R^WT^ (black). The RMSFs of the A-loop is zoomed in the insert. The average conformations for CSF-1R^WT^ (**C**) and CSF-1R^MU^ (**D**) are presented as tubes. The size of the tube is proportional to the by-residue atomic fluctuations computed on the backbone atoms. The high fluctuation region found in proteins, are specified by red color and numerated from 1 to 10 in B–D. The size of numbers in **D** is proportional to RMSFs.

Molecular dynamics (MD) simulations of the generated models (two 50-ns trajectories for each form) were carried out to investigate and compare the structure and internal dynamics of the two proteins, CSF-1R^WT^ and CSF-1R^MU^. The global dynamical behavior of the proteins was explored by measuring the root mean square deviations (RMSDs) of backbone atoms with respect to the initial frame plotted versus simulation time and showed separately for N- and C-lobes, the JMR and the A-loop regions (**Fig. 2A**). The four trajectories of CSF-1R (two replicas for CSF-1R^WT^ and two for CSF-1R^MU^) displayed comparable conformational drifts, with RMSD mean values in the range 0.12–0.30 nm indicating a tolerable stability of the simulated systems after a 5 ns relaxation interval. However, the RMSD profile of the A-loop region showed high deviation after 17 ns for one CSF-1R^MU^ replica, with RMSD values up to ∼0.26 nm, which was not observed in the other trajectories. We observed a similar behavior for the A-loop in KIT MD simulations [31], although the deviations were significantly larger than in CSF-1R.

The root mean square fluctuation (RMSF) values, describing atomic fluctuations averaged over the protein residues, ranged from 0.1 to 0.4 nm, and were overall quantitatively comparable between CSF-1R^WT^ and CSF-1R^MU^ (**Fig. 2B**). Projection of RMSF values on the tridimensional structure of CSF-1R (**Fig. 2C**) revealed that the most flexible residues formed clusters located in the JMR, encompassing the most buried JM-B fragment (residues 543–545) and part of the JM-S (residues 556–560), the A-loop, the KID, and the loop that connects β3-strand (residues 620–625) and Cα-helix in the N-lobe. The D802V mutation noticeably enhanced RMSF fluctuations in these regions (**Fig. 2D**). A zooming on the A-loop RMSF values indicated the perturbation on the atomic coordinates observed in one of the MD simulations of CSF-1R^MU^ (**Fig. 2B**, insert).

**Figure 3:**
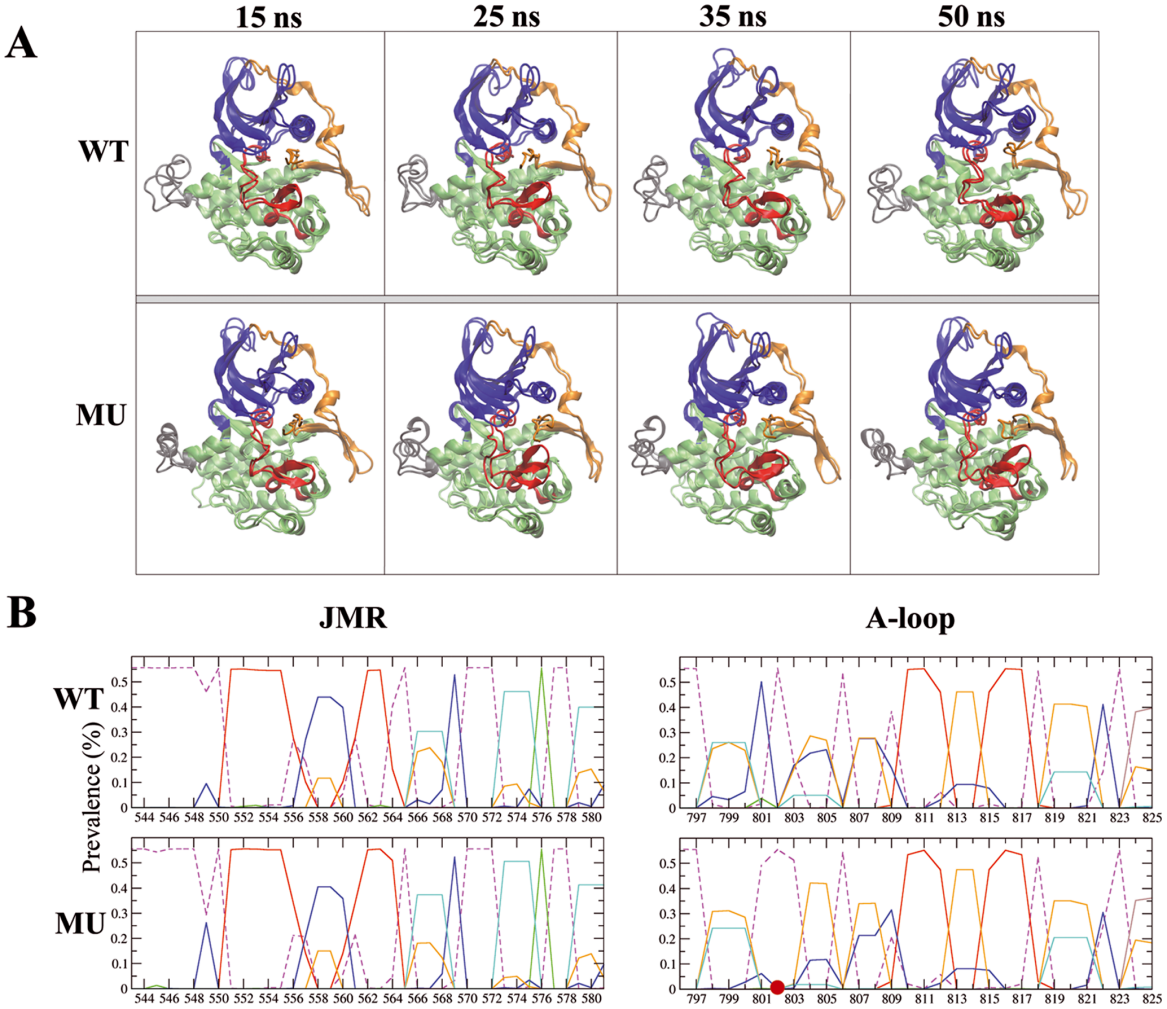
MD conformations of CSF-1R cytoplasmic region in the native protein and its D802V mutant. (**A**) Ribbon diagrams display the proteins regions or fragments with different colors: JMR (orange), A-loop (red), N- and C-lobe (blue and green), and KID (gray). Snapshots taken from the two MD replicas at 15, 25, 35 and 50 ns for CSF-1R^WT^ (top) and CSF-1R^MU^ (bottom) were superimposed by pair. Superposed conformations were selected by RMSDs clustering. (**B**) Secondary structure assignments for JMR (on the left) and A-loop (on the right) were averaged over the two 50-ns MD simulations of CSF-1R^WT^ and CSF-1R^MU^. For each residue, the proportion of every secondary structure type is given as a percentage of the total simulation time. Each secondary structure type is shown with lines of different colors: 3_10_ helices (in cyan), parallel β-sheet (in red), turn (in orange), bend (in blue) and bridge (green). Coiled structure is sown by dashed lines. The D802V position is indicated as a red circle.

Systematic analysis of the MD conformations indicated that the structure of CSF-1R cytoplasmic region was globally conserved over the simulation time in CSF-1R^WT^ and CSF-1R^MU^ and shows in general a similarity between these two forms (**Fig. 3A**). Nevertheless, a detailed inspection of the secondary structures showed different folding of the A-loop in the two proteins. The crystallographic data of the native receptor (PDB id: 2OGV) [22] show that residue D802 is located in a short bend between two small 3_10_-helices formed by residues 798–800 (*H1*) and 803–805 (*H2*). Over the MD simulations of CSF-1R^WT^ the structure of *H1* region was mainly folded as a 3_10_-helix while the *H2* region secondary structure type alternated between 3_10_-helix (5%), bend (20%), turn (30%) and coil (45%) (**Fig. 3B**, on the right). In CSF-1R^MU^, the only secondary structure element retained over the simulations is the 3_10_-helix *H1* positioned prior the D802V mutation site. The second 3_10_-helix, *H2*, which follows the mutated site, is disappeared, and the residues 803–805 adopt a turn conformation as was evidenced for most of the simulation time.

Such disappearing of the well-ordered structural element, previously observed in KIT^WT^, and the increased atomic fluctuations in the A-loop, results from the replacement of the negative capping aspartate by a hydrophobic valine, *i.e.*, the absence of the negatively charged side chain of aspartate disrupted a positive dipole moment formed by the small 3_10_-helix adjacent to the mutation, which is supposed to destabilize the inactive structure of the A-loop [78]. A similar local structural effect was observed experimentally in KIT^D816H^ (3G0F) [27] and predicted by *in silico* studies in KIT^D816V^ [31] and in KIT^D816H/N/Y^ mutants (our data submitted to publishing). The disappearance of the *H2* 3_10_-helix changed the local H-bond network in the A-loop of CSF-1R^MU^ (data not shown) as it was observed in KIT^MU^.

Whereas KIT D816V/H/N/Y mutations led systematically to a global structural reorganization of the JMR which adopts a well-shaped anti-parallel β-sheet structure translated in the axial position respectively to the KD [31], such a long-range effect, surprisingly, was not observed in CSF-1R^MU^. The JMR structure and dynamics were strikingly similar in CSF-1R^WT^ and CSF-1R^MU^. The quantitative analysis of the secondary structure pattern over the MD simulations revealed a retained secondary structure of the JMR in CSF-1R^MU^ compared to CSF-1R^WT^ (**Fig. 3B**). Moreover, despite a topical increase of the JM-B fluctuations in CSF-1R^MU^, the JMR position was rigorously maintained relative to the KD (**Fig. 3A**). On the contrary to KIT^WT^, the JMR of CSF-1R^WT^ is already folded as a well-shaped anti-parallel β-sheet, as evidenced in the crystallographic structure [22].

Altogether, KIT D816V and the homologous CSF-1R D802V similarly affect the receptor structure alone at the proximity of the mutated residue, while the JMR structure is only altered in KIT mutant. Such a difference, which can be related to the distinct sequence of these regions in the two receptors, may have functional consequences.

To explore the secondary structure profile of CSF-1R JMR (residues 538–580), we used six sequence-based secondary structure prediction methods and one structure knowledge-based method. Predictions indicated a relatively high probability of the polypeptide organization in well-folded structural elements, particularly β-strands in the segments 551–555 and 563–564 linked by a random coil including 4 residues, probably stabilized as a turn (**Fig. S2**). This secondary structure prediction matches well with the JMR structure of the native receptor (CSF-1R^WT^) observed by X-ray crystallography and obtained by MD simulations of CSF-1R^WT^ and CSF-1R^MU^. This observation prompts to hypothesize that either the JMR structure in CSF-1R does not depend on the KD − a behavior quite different from the allosterically regulated JMR folding in KIT, − or D802 in CSF-1R and D816 in KIT do not play a similar role in the activation mechanisms.

### Dynamic behavior of receptors

To address the CSF-1R structural properties related to its functions, particularly to distinguish the receptor features associated to the activation mechanisms, we characterized the dynamical behavior of both proteins, CSF-1R^WT^ and CSF-1R^MU^. We used the large-amplitude collective motions that describe the protein functional dynamics [79]. Among these motions, the most probable ones, also known as the softest modes, are usually highly collective, *i.e.*, they drive the cooperative motions of entire domains/subunits.

Here, we used the Principal Component Analysis (PCA) (i) to clarify the mutation effects in the context of collective motions between functional CSF-1R fragments in the cytoplasmic region, (ii) to compare the impact of mutation on dynamical features of CSF-1R and KIT, and (iii) to connect motions with communications between spatially distant regulatory fragments, namely A-loop and JMR. The most relevant movements of CSF-1R fragments were identified by emphasizing the amplitudes (eigenvalues) and directions (eigenvectors) of the protein motions dominating the residue pair covariance matrix calculated from the MD ensemble. The calculation was done for the individual MD simulation trajectories of each model and the best overlap between CSF-1R^WT^ and CSF-1R^MU^ was used for illustration.

**Figure 4:**
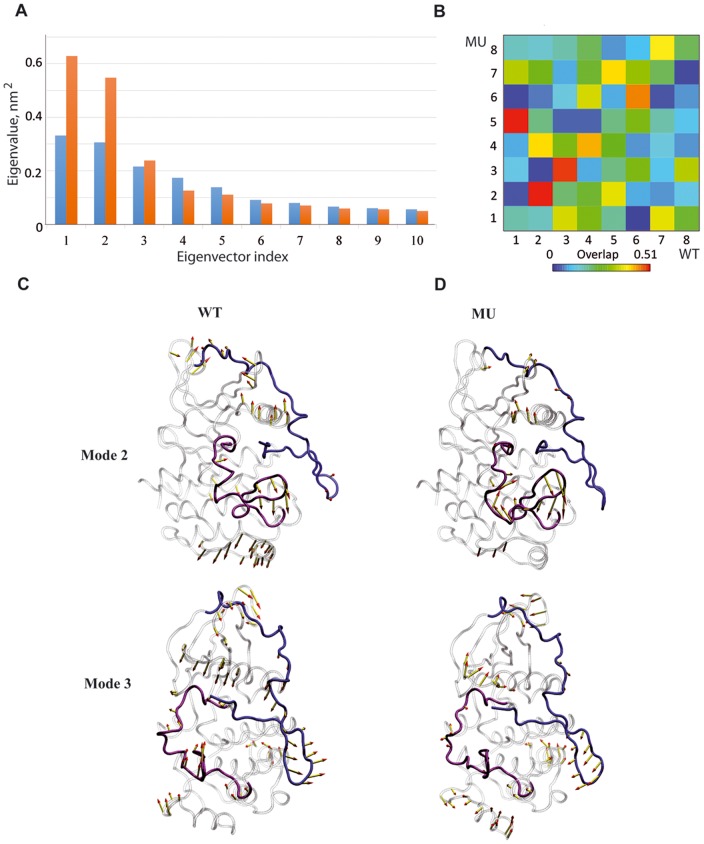
Principal component analysis (PCA) of CSF-1R cytoplasmic region in the inactive state. The calculation was performed on the backbone atoms of CSF-1R^WT^and CSF-1R^MU^. *Top*: (**A**) The barplot gives the eigenvalues spectra of CSF-1R^WT^(blue) and CSF-1R^MU^ (orange) in descending order. (**B**) The grid gives the overlap between the first 10 eigenvectors from CSF-1R^WT^ (columns) and CSF-1R^MU^ (rows). The overlap between two eigenvectors is evaluated as their scalar product and represented by a colored rectangles, from blue (0) through green and yellow to red (0.51). *Bottom*: Modes 2 and 3 atomic components for CSF-1R^WT^ (**C**) and CSF-1R^MU^ (**D**) are drawn as yellow arrows on the protein cartoon representation. JMR is in blue, A-loop is in violet and the rest of protein is in grey.

Among the first 10 eigenvectors, which contribute the most to the total atomic fluctuations, the first two modes of CSF-1R^MU^ display eigenvalues twice as big as those of CSF-1R^WT^ (**Fig. 4A**). The overlap between the eigenvectors showed a good agreement between CSF-1R^WT^ and CSF-1R^MU^ for modes 2 and 3 (**Fig. 4B**). In both CSF-1R^WT^ and CSF-1R^MU^, the 2^nd^ mode was associated mainly with the displacement of the A-loop, the loop linking β3-strand and Cα-helix and the C-terminus. Mode 3 showed the concerted movements of the loops connecting the β-sheet in the N-lobe and also movements in the proximity of the C-terminus, while we did not observed any movement in the KD correlated to the JMR motions in both receptors. Noticeably the observed JMS motions in mode 3 depict “back-and-forward” movements in both models, which are not characteristic of JMR departure (**Fig. 4C–D**).

Normal modes analyses (NMA) were carried out to further characterize the collective movements related to the JMR. The choice of the initial CSF-1R conformations was based on a convergence analysis performed on the merged trajectories [61]. The stability of the systems was described in terms of representative MD conformations. Briefly, a set of *reference* structures were picked up randomly among the MD conformational ensemble of the trajectories and *reference* groups were composed of conformations from the two replicas of each trajectory. A good convergence quality can be assessed when each *reference* structure is more or less equally represented in both replicas. A *lone reference* structure is defined as a *reference* structure that is not represented in one-half of the trajectory (one empty *reference* group). To ensure the robustness of the method, we performed the analyses using five different random seeds for the *reference* structure picking up. For each form of the receptors, the fourth run containing the set of conformations that was better represented among the different replicas was chosen.

The results of this analysis are summarized in **Table S1** and **Fig. S3**. The computed degrees of collectivity of JMR atomic motions, *k_JMR_*, ranged from *1/n* (only one atom among *n* is involved in the motion) to 1 (highly collective). The mean *k_JMR_* value for CSF-1R^WT^ and CSF-1R^MU^ of 0.44 and 0.42 respectively, indicating a low and statistically identical degree of collectivity in both proteins denoting the absence of independent motions associated with the JMR. The modes correlated to movements located at the JMR clearly indicated a great similarity between CSF-1R^WT^ and CSF-1R^MU^. Altogether, the NM analysis confirmed the absence of JMR displacement from the KD in the mutated protein, evidenced by the PCA.

### Coupling between JMR and KD in receptors

**Figure 5:**
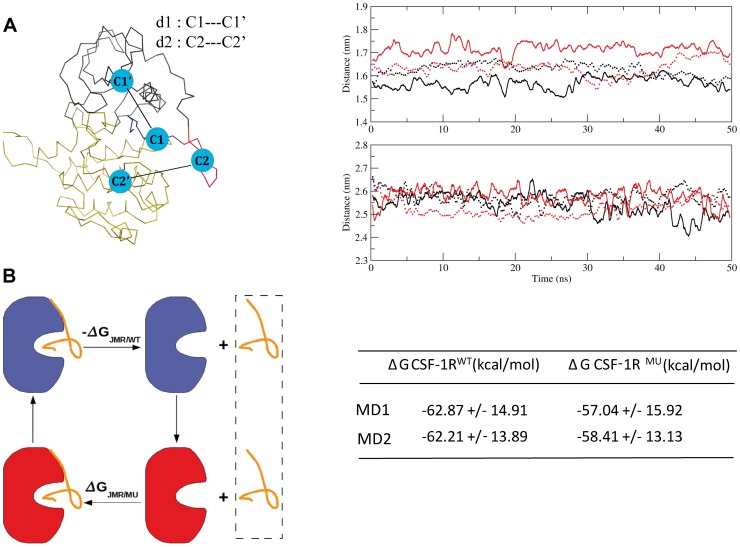
Protein stability and binding energy changes between CSF-1R^WT^and CSF-1R^MU^ in the inactive state. (A) *Left* : Distances d1 and d2 between the centroids C1 (JM-B)) and C1′ (N-lobe) and between C2 (JM-S) and C2′ (C-lobe), respectively. Right : Distance d1 (at the top) and d2 (at the bottom) monitored during the two replicas of the 50 ns MD simulations (full and dashed lines) for CSF-1R^WT^ (black) and CSF-1R^MU^ (red). (B) Left : A thermodynamic cycle picturing the dissociation of JMR from KD in CSF-1R^WT^and CSF-1R^MU^. Right: The total free energy (ΔG) of the JMR binding to the kinase domain, computed over the individual MD simulations for both CSF-1R^WT^and CSF-1R^MU^.

In order to probe a possible coupling of the JMR with the kinase domain (KD), we first characterized the relative position of these two receptor's portions using two geometrical parameters, d1 and d2, describing the distance between the centroids defined on JM-B and N-lobe, and JM-S and C-lobe, respectively (**Fig. 5A**). Monitoring of these distances over the MD simulations indicated a very slight increase (∼0.11 nm) of d1 from the initial value observed in only one MD trajectory of CSF-1R^MU^. The d2 profiles of the two proteins blend into each other, demonstrating that JM-S and C-lobe retained their relative position in the mutated receptor. Secondly, we calculated the binding energy associated to the interaction between JMR and KD. The free energy of binding (ΔG) computed over the individual MD simulations by the MMGBSA method showed a tendency of JMR to display a lower affinity with the KD in CSF-1R^MU^ than in CSF-1R^WT^ (**Fig. 5B**), similarly to previous observations in KIT^MU^ [31]. Apparently, this difference was more pronounced in KIT than in CSF-1R, indicating a stronger coupling of JMR and KD in CSF-1R. Such a coupling stabilizes the overall protein structure and dynamical behavior evidenced by the low amplitude of the motions/fluctuations of JMR.

**Figure 6:**
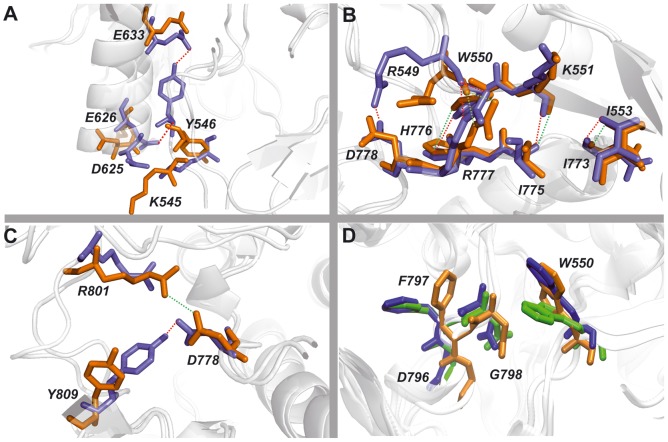
H-bond patterns in CSF-1R stabilising the auto-inhibited inactive state of CSF-1R^WT^and the non-inhibited inactive state of CSF-1R^MU^. H-bonds between residues from (A) JMR and Cα-helix; (B)JMR and C- loop and (C) A-loop and C-loop. Snapshots taken from the most representative conformations derived from MD simulations by the convergence analysis. All residues presented as sticks, in blue for CSF-1R^WT^and in orange for CSF-1R^MU^. The H-bonds are shown as dotted lines, red and green in CSF-1R^WT^and CSF-1R^MU^ respectively. (D) The DFG motif conformation together with JMR's anchoring residue W550. Representation of DFG and W550 residues conformations originated from the crystallographic structure (2OGV, green) and representative MD conformations of CSF-1R^WT^ (blue) and CSF-1R^MU^ (orange).

We also used the MD simulations data to calculate the occurrences of H-bonds involving key residues that maintain the inactive auto-inhibited form of CSF-1R [22]. The H-bonds describing the contacts of the JMR and the A-loop residues with the residues from N- and C-lobes are summarized in **Table 1** and illustrated in **Fig. 6**.

**Table 1:** H-bonds stabilized the inactive conformation in CSF-1R^WT^ and CSF1R^MU^.

**JMR − C-helix contacts**			**A-loop − C-lobe contacts**		
H-bond	CSF-1R^WT^	CSF-1R^MU^	H-bond	CSF-1R^WT^	CSF-1R^MU^
Y546•••E633	82*	19*	E825•••R900	100	100
K545•••D625	79	35	W821•••E847	100	100
Y546•••E626	68	38	W821•••S840	99	98
K545•••E628	-	30	Y809•••D778	82	42
T567•••K635	43	46	E825•••S636	79	68
K543•••E636	13	-	K820•••R855	63	61
Y546•••D625	-	30	R801•••R782	58	38
**JMR− C-lobe contacts**			D806•••R782	48	-
H-bond	CSF-1R^WT^	CSF-1R^MU^	K820•••N854	46	35
I553•••N773	100	100	Y809•••R782	44	33
R549•••R777	100	100	R801•••N783	34	26
K551•••I775	100	100	N808•••N854	20	29
R549•••D778	57	-	R801•••N778	17*	82*
W550•••H776	54	38	P797•••N783	17	-
Y556•••V834	-	37			
Y556•••N773	21	21			
Y556•••Q835	-	20			

Residues involved in H-bonding and the H-bond occurrences (in %) are computed over MD simulations. Occurrences showed a major difference are denoted by asterisk.

The relative position of JM-B and KD residues in CSF-1R^MU^ appeared to be unfavorable to the H-bonds pattern (**Fig. 6A**). The occurrence of key H-bonds contributing to JMR anchoring to the KD, and to A-loop maintenance in an inactive conformation, were dramatically reduced in CSF-1R^MU^ (**Table 1**). The interaction between the JMR and the N-lobe, which is stabilized by an H-bond between Y546 (JM-B) and E633 (Cα-helix), was reduced by a factor of 4 in CSF-1R^MU^ compared to CSF-1R^WT^. The occurrence of two other H-bonds, K545•••D625 and Y546•••E626, was reduced by a factor 2 in CSF-1R^MU^ compared to CSF-1R^WT^. An alternative H-bond involving Y546 and D625 was detected in CSF-1R^MU^, suggesting a partial compensatory effect.

Conversely, the H-bonds between the JMR and the catalytic loop from the C-lobe in CSF-1R^MU^ display none or only slight changes respectively to CSF-1R^WT^ (**Table 1**, **Fig. 6B**). This observation indicates the strong coupling between the JMR and the C-lobe in both forms of receptor and correlates well with the highly conserved position of JMR respectively to kinase domain in CSF-1R^WT^ and CSF-1R^MU^.

In addition to Y546, W550 is a crucial JM-B anchoring residue [22] that helps to hinder the active conformation of the A-loop by occupying the position that F797 (DFG motif) would acquire in the active form [21]. Representative structures derived from MD simulations showed a displacement of W550 side chain away from the ATP-binding site in CSF-1R^WT^ and CSF-1R^MU^, when compared to its position in the crystallographic structure (**Fig. 6C**). Remarkably, the DFG motif in CSF-1R^MU^ shows a conformational change in respect to CSF-1R^WT^ in the crystal and in the MD conformations (**Fig. 6D**). All residues of the DFG motif in CSF-1R^MU^ are slightly displaced from their positions in CSF-1R^WT^, and F797 side chain is pointed away from the ATP-binding site. Such position of F797 described as an “in” conformation the DFG motif that is specific for the inactive non-autoinhibited conformation of the receptor. The highly conserved residue F797, appears to serve as a conformational switch in the receptor.

The A-loop inactive conformation was also stabilized by interaction of Y809 (A-loop) as a pseudo-substrate with the catalytic loop residue D778 (C-lobe) in CSF-1R^WT^ through the H-bond Y809•••D778, which is decreased by a factor of 2 in CSF-1R^MU^. This destabilizing effect in mutant is compensated by H-bond R801•••D778, favored by the displacement of the R801 towards D778 (**Fig. 6C**, **Table 1** **)**.

Further, we compared the electrostatic potential surfaces of JMR and kinase domain in both receptors. The calculations were performed by the Adaptive Poisson-Boltzmann Solver (APBS) software using the crystallographic structures describing the inactive auto-inhibited state of the native receptors, CSF-1R (PDB id: 2OGV, [22] and KIT 1T45, [21]) receptors.

**Figure 7:**
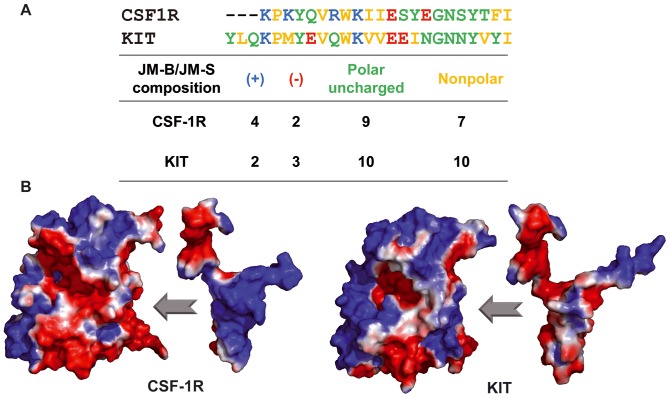
Features of the JMR sequence in CSF-1R and KIT and Electrostatic Potential (EP) surface in the two receptors. (**A**) The amino acids composition of JMR (JM-B and JM-S) in CSF-1R and KIT. (**B**) The EP surface of KD and JMR in two receptors, CSF-1R and KIT. EP calculations on the Connolly solvent-accessible surfaces of the receptors were performed with the APBS software. The color scale ranges from red (electronegative potential) through white (neutral) to blue (electropositive potential).

Although their structure is very similar, the two receptors display important sequence divergence in JMR (**Fig. 1**) and KID. The JMR sequence contains 50 residues in KIT and 43 residues in CSF1R, including 8 basic residues in CSF1R versus 6 in KIT; the number of polar residues is more significant in CSF1R (18) than in KIT (15) (**Fig. 7A**). These subtle differences alter significantly the electrostatic surface of both proteins. Particularly in CSF-1R, the charge complementarity between the JMR and the KD surfaces favors direct contacts between them (**Fig. 7B**). Such profile in KIT shows relatively limited complementarity between the JMR and KD surfaces. The different profiles of the electrostatic potential surfaces in the two receptors are derived from the different nature of the amino acids compositions of this region, principally in JMR. The strong Coulomb interactions and the relevant H-bonds occurrences between JMR and kinase domain in CSF-1R indicate the tight coupling of these functional domains.

### Communication pathways in receptors and they functional role

The JMR coupling with kinase domain controls the receptor activation process. It has been described previously that allosteric coupling can be mediated solely by transmitted changes in the protein dynamics/motions as a consequence of a re-distribution of the protein conformational populations [76], [80]–[83]. We recently developed a novel method, the MOdular NETwork Analysis (MONETA), designed for accurate characterization of communication pathways in a protein by exploring the inter-residues dynamical correlations computed from MD trajectories and the intramolecular non-bonded interactions [32]. Such approach applied to KIT put in evidence a well-established communication between the JMR and the A-loop tyrosine Y823 in KIT^WT^, manifested as an extended network of H-bonds linking these two remote regions, the JMR and the A-loop, through the catalytic loop. D792 and Y823, linked in KIT^WT^ by a strong and stable H-bond, were identified as key residues in establishing of communication pathways. Destruction of this H-bond in KIT^MU^ interrupted the allosteric coupling between these receptor segments leading to the structural changes in the JMR of KIT^D816V^.

A study of CSF-1R using MONETA was performed to (i) analyze the communication pathways in the cytoplasmic domain of the receptor, (ii) evaluate the role of residue D802 in communication pathways and finally (iii) assess the impact of the D802V mutation on the protein internal communication network.

Identification of the protein regions representing the most striking local features of the two proteins' internal dynamics was carried out using a statistical technique known as Local Feature Analysis (LFA) [74] adapted from image processing to proteins [84]. This method identifies clusters of residues named *Independent Dynamic Segments* (*IDSs*) that are formed around each *seed* and display concerted local atomic fluctuations and independent dynamical behavior [32].

The number of PCA modes retained for LFA was 17 in CSF-1R^WT^ and 19 in CSF-1R^MU^, the number of *IDSs* identified by MONETA being 8 in CSF-1R^WT^ and 9 in CSF-1R^MU^, respectively. The *IDSs* differences between the two receptors concern their feature, location, and size. To optimize the comparative analysis, the distinct *IDSs* were referred to as S_i_, where i = 1,2,…,N.

**Figure 8:**
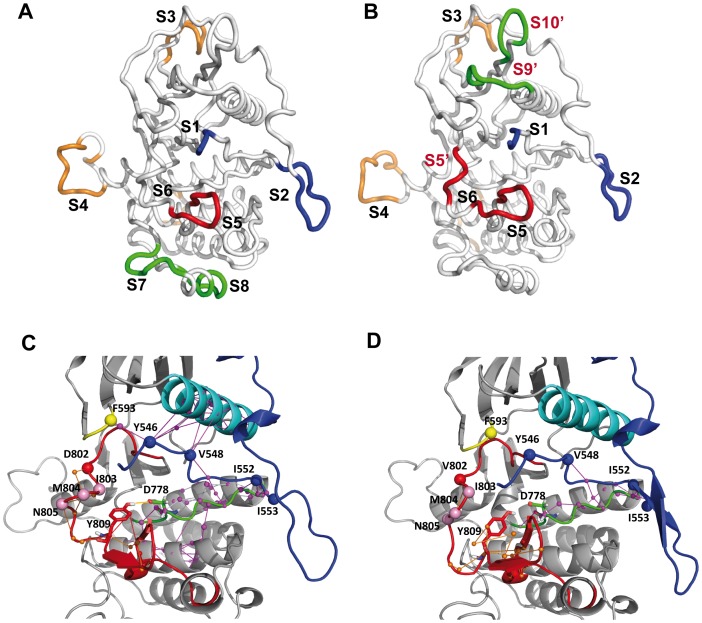
Independent dynamic segments and communication pathways in cytoplasmic region of CSF-1R. *Top*: Structural mapping of the *Independent Dynamic Segments* (*IDSs*) identified in CSF-1R^WT^ (**A**) and CSF-1R^MU^ (**B**). The average conformations are presented as tubes. *IDSs* were localized from the analysis of the merged 60 ns concatenated trajectory. *IDSs* are referred to as *S_i_*, where *i* = 1, 2,…,N, labeled and specified by color retained for the *IDSs* in the both proteins. The largely modified or newly found *IDSs* in the mutant are referred to as *S′_i_* in red. *Bottom*: 3D structural mapping of the inter-residues communication in CSF-1R^WT^ (**C**) and CSF-1R^MU^ (**D**), computed over the last 30 ns of the individual MD simulations. MD 2 is taken for illustration. The average MD conformation is presented as cartoon. The proteins fragments are presented with different colors: JMR (blue), Cα-helix (cyan), P-loop (yellow), C-loop (green) and A-loop (red). C*ommunication pathways* (*CPs*) between residues atoms (small circles) are depicted by coloured lines: *CPs* formed by the A-loop residues in orange; by the JMR-residues in magenta. The key residues in the communication networks are labelled (in CSF-1R^WT^) and depicted as bulky circles.

*IDSs* common to the two forms of receptor are located in JM-B (S1, residues 543–546), in JM-S (S2, residues 553–562 in CSF-1R^WT^ and 554–562 in CSF-1R^MU^), in the solvent-exposed loop that connects β2 and β3 (S3, residues 602–611) in the N-lobe, in the pseudo-KID (S4, residues 678–692), in the A-loop (S5, residues 810–817 in CSF-1R^WT^ and 809–817 in CSF-1R^MU^), and in the C-terminal tail (S6, residues 914–922) (**Fig. 8 A,B**). The two *IDSs* specifically observed in CSF-1R^WT^ were found in the C-lobe (S7, residues 856–862 of the loop that connects Hα- and Gα-helices; S8, residues 867–874 in the G-helix). The three *IDSs* specifically observed in CSF-1R^MU^ were localized in the N-lobe (S9′, residues 617–624 of the loop that connects β3 and Cα-helix; S10′, residues 654–659 in the loop linking β4 and β5) and in the A-loop (S5′, residues 802–806). Interestingly, the residues forming S9′ in CSF-1R^MU^ were also found in S1, suggesting that the JM-B and the loop linking β3 and Cα-helix were associated in an entire self-reliant *IDS* (not shown). The other unexpected observations were the participation of D802V and Y809 in S5′ and S5, respectively.

Using MONETA, we identified only one *IDS* in the N-lobe of CSF-1R^WT^ and three in that of KIT^WT^ [32], whereas *IDSs* in the JMR, the A-loop, the pseudo-KID, and the G-helix were identical in the two native receptors. The impact of the equivalent mutation on the *IDSs* in the cytoplasmic region of the two receptors is dissimilar. In CSF-1R^MU^ three novel *IDSs*, S5′, S9′ and S10′, are a consequence of increased concerted local motions of the A-loop and the loops linking β3 with Cα-helix, and β4 with β5 (**Fig. 2**). In KIT^MU^ such motion increase was observed only at the A-loop; the motions in two other loops were diminished respectively to KIT^WT^ [31]. The two A-loop *IDSs*, S5 and S5′, separated in CSF-1R^MU^, were observed as superimposed and duplicated *IDSs* in KIT^MU^ [32]. The two key residues, the point mutation and the A-loop tyrosine, are involved in *IDSs* (S5′ and S5 respectively) in CSF-1R^MU^, while in KIT^MU^, only the point mutation is located in *IDS*.

Further, we studied the inter-residue communications linking different *IDSs*. To quantify the inter-residues communications, we computed the number of *communication pathways* (*CPs*) for each protein. In virtue of the strong influence of the dynamical behavior onto the communication pathways, the calculation of *CPs* was performed based on the individual MD simulations. For instance, the communication network computed over the 60 ns concatenated trajectory contains 1692 and 1626 non-redundant paths in CSF-1R^WT^ and CSF-1R^MU^ respectively, indicating the mutation-induced diminishing of the communication network in the receptor (**Table 2**). Nevertheless, the total number of *CPs* can vary considerably among the different replicas for both forms.

**Table 2:** Quantitative analysis of the communication network pattern among the different MD replicas.

Parameter	CSF-1R^WT^			CSF-1R^MU^		
	MD 1	MD 2	MD 12	MD 1	MD 2	MD 12
Shortest paths*	2082	2953	1692	2679	2341	1626
Hubs	39	66	30	57	48	36
Number of paths derived from A-loop residues						
D/V802	1	1		1	1	
Y809	3	5		5	9	
Number of shortest paths* connecting JMR to other functional segments						
JM-B− P-loop	1	1		0	0	
JM-B− Cα helix	0	17		3	1	
JMR− C-loop	24	27		39	21	

MD1, MD2 and MD12 are the two separate and merged trajectories respectively.

*Shortest paths  =  smallest paths involving two residues [73].

We were interested to investigate if the mutation D802V would compromise the communication between the receptor fragments determined as crucial in the activation mechanisms. Therefore, we looked for the *CPs* derived from the mutation site D(V)802, the A-loop tyrosine Y809 and the *CPs* that connect JMR residues to other functional TKD segments, such as the P-loop, the Cα-helix and the C-loop, all involved in the stabilization of the inactive auto-inhibited conformation of the JMR (**Table 2**).

Despite a variation of the number of paths and their communication profile among the two replicas for the same system, the data characterizing different forms of receptor indicate that the JMR communication, especially when involving the JM-B, is considerably affected in CSF-1R^MU^ respective to CSF-1R^WT^. These data suggests that a local perturbation on the A-loop affects the JM-B communication with the P-loop and the Cα-helix, although JMR residues maintained a strong communication with the C-lobe, through the C-loop.

The differences in communication are illustrated using replica MD 2 for both CSF-1R^WT^ and CSF-1R^MU^. The *communication pathways* identified by MONETA form either local small *CP* clusters or extended networks (**Fig. 8 C–D**). In CSF-1R^WT^, D802 is involved only in a local *CP* protruded to M804 in the small 3_10_-helix *H2* of A-loop, posterior to the mutation site. Y809 initiated short *CPs* with other A-loop residues, particularly with S807, L817, P818, V819 and W821. Similarly, to KIT^WT^, no direct *CP* between the JMR and the A-loop in CSF-1R^WT^ was identified. Nevertheless, the side chain of Y809 points toward the C-loop, probably as an effect of the H-bond Y809•••D778, highly prevalent during the MD simulations (**Table 1**). Moreover, D778 in the C-loop is involved in a *CP* extended toward the JMR (**Fig. 8 C**). Consequently, this *CP* can transmit information from the JM-S residues forming *IDS* S2 to the catalytic (C-) loop residue D778, and further, through the H-bond Y809•••D778, to the A-loop residues. The JM-S residues are involved in distinct *CP* networks providing connection of the JMR to the other functionally crucial fragments of the kinase domain.

The well-established *communication pathways* formed by the JM-B residues (Y546 and V548) with the P-loop (F593) and the Cα-helix (residues 628–633), the extended *CPs* from the JMR residues reaching the C-loop, and the Eα- Fα- and Hα-helices, constitute a developed multi-branched *CP* network capable to coordinate the movements of N- and C-lobes involved in CSF-1R activation mechanisms, *i.e.* post-translational modifications and catalytic functions. Interestingly, the *CPs* of each α-helix, Cα, Eα, Fα and Hα, are extended over the entire helix length, making a structurally preformed *communication fiber*. A considerable part of this extended *CP* network is completely lost in MD 2 from CSF-1R^MU^, *i.e.*, no *CP* was observed between the JM-B and the P-loop, the Cα-, or the Hα-helices. Nevertheless, a relatively extended *CP* network is still observed between the JMR and the C-loop and the Eα- helix in CSF-1R^MU^ (**Fig.8 D**). This remaining network establishes communication between D778 and the JM-Switch but do not extend to the A-loop. Indeed, the H-bond Y809•••D778 controlling such CP extension in CSF-1R^WT^, shows a two-fold diminished prevalence in CSF-1R^MU^.

We also evidenced that, in CSF-1R, *communication pathways* connect S1 (JM-Binder) and S2 (JM-Switch) mainly to the molecular fragments not manifesting the concerted local atomic fluctuations (*IDSs*), except S5 formed by residues from the A-loop β-sheets. The links between residues belonging to *IDSs* and the other receptor fragments involved in *CPs* are held by H-bonds (**Table 1**). In CSF-1R^MU^, the absence of H-bonds between the JM-B and the Cα-helix residues significantly altered *CP* profiles. Diminished occurrence of the H-bond Y809•••D778 provokes the *CP* interruption between V802 and Y809 which in CSF-1R^MU^ are involved in S5 and S5′ *IDSs* respectively. By contrast, the conserved H-bond pattern between the JMR residues involved in S1 and S2 *IDSs* and the catalytic loop partially preserves the *CP* that links these *IDSs* with the C-lobe residues similarly to CSF-1R^WT^.

Our analysis showed that despite a comparable pattern of *CPs* between the JMR and the A-loop in CSF-1R and KIT, their functional roles appear to be different. The established *CP* between the A-loop and the JMR through the catalytic (C-) loop is crucial for maintaining the allosteric regulation of the KD in KIT and its disruption in KIT^MU^ is a major contribution to its constitutive activation [32].

Another particularity of the CSF-1R communication pattern consists of the JMR communication with the glycine-rich P-loop and with the Cα-helix, not observed in KIT (**Fig. S4**). Mutual *CPs* of the JM-B residues with the Cα-helix are extended over the entire helix length in the native protein, while few and relatively small *CPs* are observed in KIT.

To search the origin of such difference in the two structurally similar receptors from the same RTKs family having a considerable sequence identity, we pointed to the structural features of these receptors. Comparative inspection of the N-terminal domain structure in both receptors evidenced that position of the P-loop and the Cα-helix is (i) equivalent in the inactive state of both receptors; (ii) conserved over the inactive-to-active forms transition in CSF-1R; and (iii) highly dissimilar in KIT active and inactive forms (**Fig. S5**). Indeed, the P-loop and the Cα-helix in the active state of KIT are shifted respectively to their positions in the inactive autoinhibited state. The relative position of the P-loop and the Cα-helix in the active and inactive forms, which is equivalent in CSF-1R and divergent KIT, may reflect their different implication in the mechanisms regulating the activation of the two receptors. This hypothesis is coherent with the different communication pathways observed in the inactive autoinhibited state of these receptors. Nevertheless, such hypothesis requires an advanced examination of the structural features of both receptors in the active state. The crystallographic structure of CSF-1R active form (PDB id: 3LCD, [85]) was stabilized by a co-crystallized kinase inhibitor, while KIT active state structure (PDB id:1PKG, [86]) was reported with two phosphorylated tyrosine residues (Y568 and Y570) and with ADP bound in the active site. These structural peculiarities suggest that displacement of the P-loop and the Cα-helix in KIT active state may be induced by phosphorylation events.

Another issue consists of the role of the allosteric communication between JMR and A-loop in CSF-1R. We evidenced early that disruption of this communication in KIT mutant provokes a structural reorganization in the JMR, distant by more than 15 Å from the point mutation. Such important structural reorganization evidenced as a folding of the β-sheet of the JMR in KIT^MU^ should induce a distinct adaptation of the phosphotyrosine-based sites which in turn may affect downstream signalling, which might not be the case in CSF-1R^MU^. As we evidenced, in the native receptors, the JMR is more attached to the kinase domain in CSF-1R than in KIT. The strong complementarities of surfaces maintain the position of JMR relative to kinase domain over the MD simulations in CSF-1R^WT^ and CSF-1R^MU^. However the atomic fluctuations of the JMR and of the Cα-helix, increased significantly in CSF-1R^MU^, suggest that the mutation-induced long-range effect is also present in CSF-1R but much more subtle than in KIT. Manifestation of this mutation-induced allosteric effect was evidenced by MONETA, revealing the disruption of communication between JMR and A-loop in CSF-1R, similarly to KIT.

## Concluding Remarks

The conformational plasticity of RTKs endows these receptors with a wide range of functions that must be tightly tuned. Gain-of-function mutations can alter this tight tuning at different levels, including ligand binding, receptor dimerization, kinase domain conformation transition, and post-translational modifications. These mutations can also trigger cell resistance to tyrosine kinase inhibitors such as Imatinib, as demonstrated for D816V in KIT and D802V in CSF-1R [78]. Such mutations might be expected to promote constitutive activation of the receptor and tumor formation [87]. Actually, KIT D816V gain-of-function mutation is a well-characterized oncogenic event and identified in more than 80% of systemic mastocytosis, whereas the equivalent CSF-1R D802V mutation has not been found in human tumors [17].

By combining various methods (MD, PCA, NMA, MONETA) to analyze and compare the structure and molecular dynamics of the native and mutated KIT and CSF-1R, the present study demonstrates that the two homologous mutations do not have the same consequences in terms of receptor conformation and dynamics, providing a plausible explanation for the differential incidence of these mutations in oncology. The local impact of D802V mutation, which is a partial unfolding of the small 3_10_-helix at proximity of the mutation site in CSF-1R, is very similar to that observed in KIT D816V [31]. Mutation-induced stabilization of the inactive non-autoinhibited conformation of both receptors, non-adapted for binding of inhibitors targeting the inactive autoinhibited state, may explain the resistance to these inhibitors.

The two mutations also disrupt the allosteric communication between two essential regulatory fragments of the receptor, the JMR and the A-loop. Nevertheless, the similarity is limited to only this effect. The mutation-induced shift towards an active conformation observed in KIT D816V is not observed in CSF-1R D802V. This differential impact on the conformational dynamics of the receptor, which might be related to differences in the primary sequence between the two wild-type receptors, particularly in the JMR region, could explain why the CSF-1R D802V does not confer a competitive advantage to the cell, thus is not retained as a driver oncogenic event. Nevertheless, it will be of interest to follow the response to new drugs, including small molecule kinase inhibitors, currently developed to target CSF-1R in cancer in which the receptor is often over-expressed, either on tumor cells or in the microenvironment [88]. Also, it is important to watch over the appearance of mutations allowing target cells to escape to the inhibitory activity of these drugs.

## Supporting Information

Figure S1 **Structural organization of RTK III receptors.** Receptor tyrosine kinases of type III comprise an extracellular cytokine binding region subdivided into five domains (from D1 to D5), a single transmembrane (TM) helix, a juxtamembrane region (JMR), a conserved tyrosine kinase (TK) domain containing a kinase insert domain (KID) and a carboxy-terminal tail. Specifically for CSF-1R, locations of mutation D802V and the main phosphorylation sites implicated in receptor activation are represented in the JMR and the activation (A-) loop.
(TIF)

Figure S2 **Secondary structure prediction of the JMR sequence (residues 538–580) from CSF-1R^WT^.** Prediction was performed using sequence-based algorithms GOR4 [33], Jpred [34], SOPMA [42], SCRATCH [44], NetSurfP [45], Psipred [46] and a structure-based method STRIDE [47]. Predicted structural elements are coded as indicated at bottom.
(TIF)

Figure S3 **Convergence analysis of the MD simulations for CSF-1R^WT^ (WT) and CSF-1R^MU^ (D802V) models performed on the 90 ns concatenated trajectories.** Grouping of MD conformations was made using five independent runs calculated for each model. The populations of each group for each run are presented as histograms in the logarithmic scale denoted by different colors, black and grey from the 1^st^ and 2^nd^ halves of the two replica respectively. The identification numbers of each reference structure denotes the time (ns) in which it was picked from the MD trajectory. The fourth run contains reference structures that are better represented in both replicas and it was chosen for further NM calculations.
(TIF)

Figure S4 **3D structural mapping of the inter-residues communication in KIT^WT^ and KIT^MU^.** The average MD conformation is presented as cartoon. The proteins fragments are presented with different colors: JMR (blue), Cα-helix (violet), P-loop (yellow), C-loop (green) and A-loop (red). C*ommunication pathways* (*CPs*) between residues atoms (small circles) are depicted by coloured lines: *CPs* formed by the A-loop residues in orange; by the JMR-residues in magenta. The key residues in the communication networks are labelled (in KIT^WT^) and depicted as bulky circles.
(TIF)

Figure S5 **Structure of the cytoplasmic domain of CSF-1R and KIT in the native form.** Superimposition of the CSF-1R and KIT crystallographic structures : (**A**) CSF-1R (2OGV [22]) and KIT (1T45 [21]) in the inactive conformation; (**B**) CSF-1R in the inactive (2OGV[22]) and the active conformations (3LCD [85]; (**C**) KIT in the inactive (1T45) and active (1PKG, [86]) conformations. The proteins are presented as cartoon, CSF-1R is in blue light and KIT is in grey light. The key structural fragments of receptors in the inactive and the active conformations are highlighted in color. The JMR is in yellow and in orange; the A-loop is in red and magenta; the Cα-helix is in cyan and blue. The relative orientation of the Cα-helix (inserts) in two proteins is presented together with the principal axis of helices detected with PyMol.
(TIF)

Table S1 **Characteristics of convergence analysis of the native CSF-1R (WT) and its mutant (D802V) MD trajectories.**
(DOC)
