# Supplementary figures and images for: Differential Effects of CSF-1R D802V and KIT D816V Homologous Mutations on Receptor Tertiary Structure and Allosteric Communication
Source: PLoS One. 2014 May 14;9(5):e97519. doi: 10.1371/journal.pone.0097519 (PMC4020833; doi:10.1371/journal.pone.0097519)

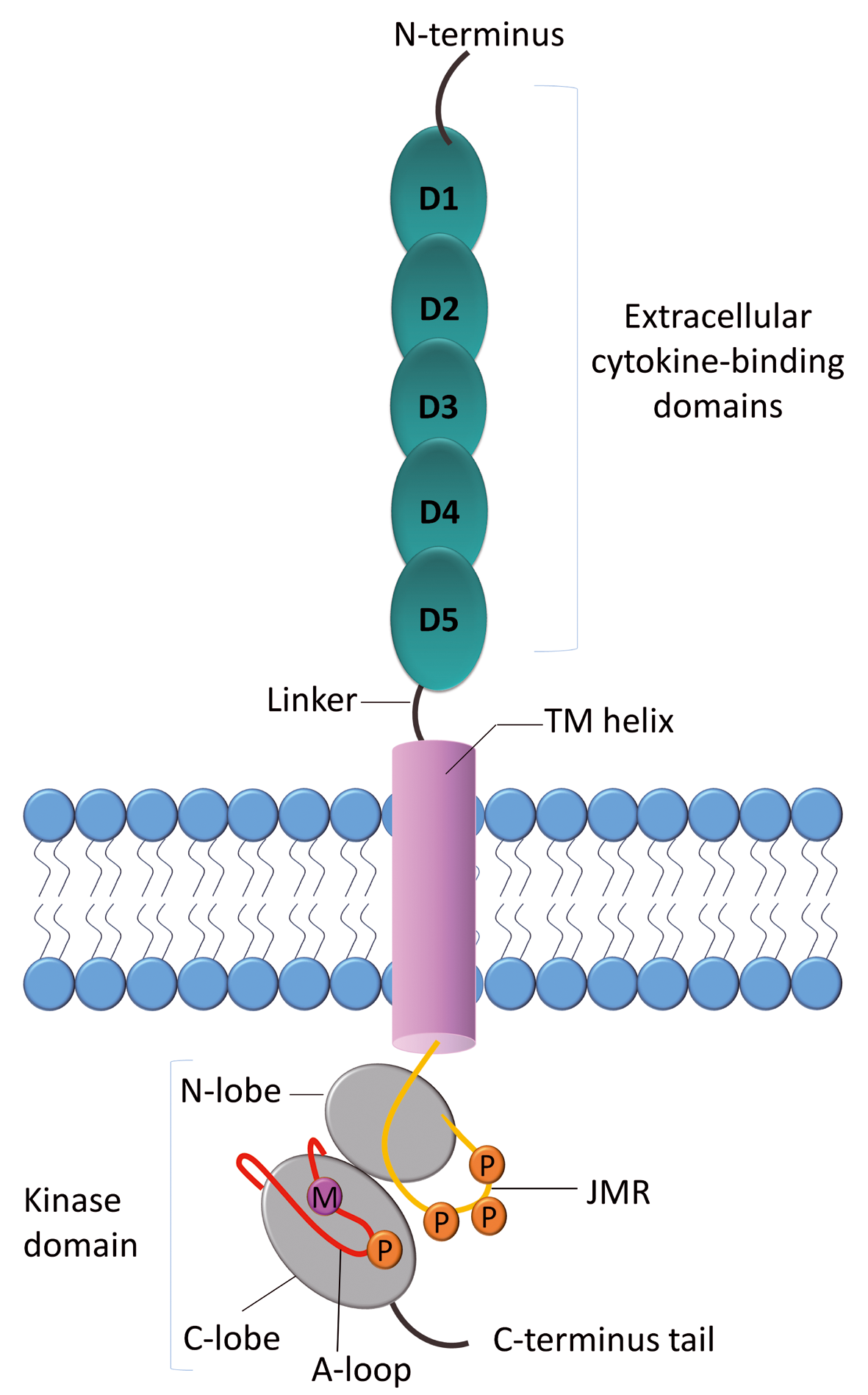

Supplement: Figure S1 — Structural organization of RTK III receptors. Receptor tyrosine kinases of type III comprise an extracellular cytokine binding region subdivided into five domains (from D1 to D5), a single transmembrane (TM) helix, a juxtamembrane region (JMR), a conserved tyrosine kinase (TK) domain containing a kinase insert domain (KID) and a carboxy-terminal tail. Specifically for CSF-1R, locations of mutation D802V and the main phosphorylation sites implicated in receptor activation are represented in the JMR and the activation (A-) loop. (TIF) [file pone.0097519.s001.tif]

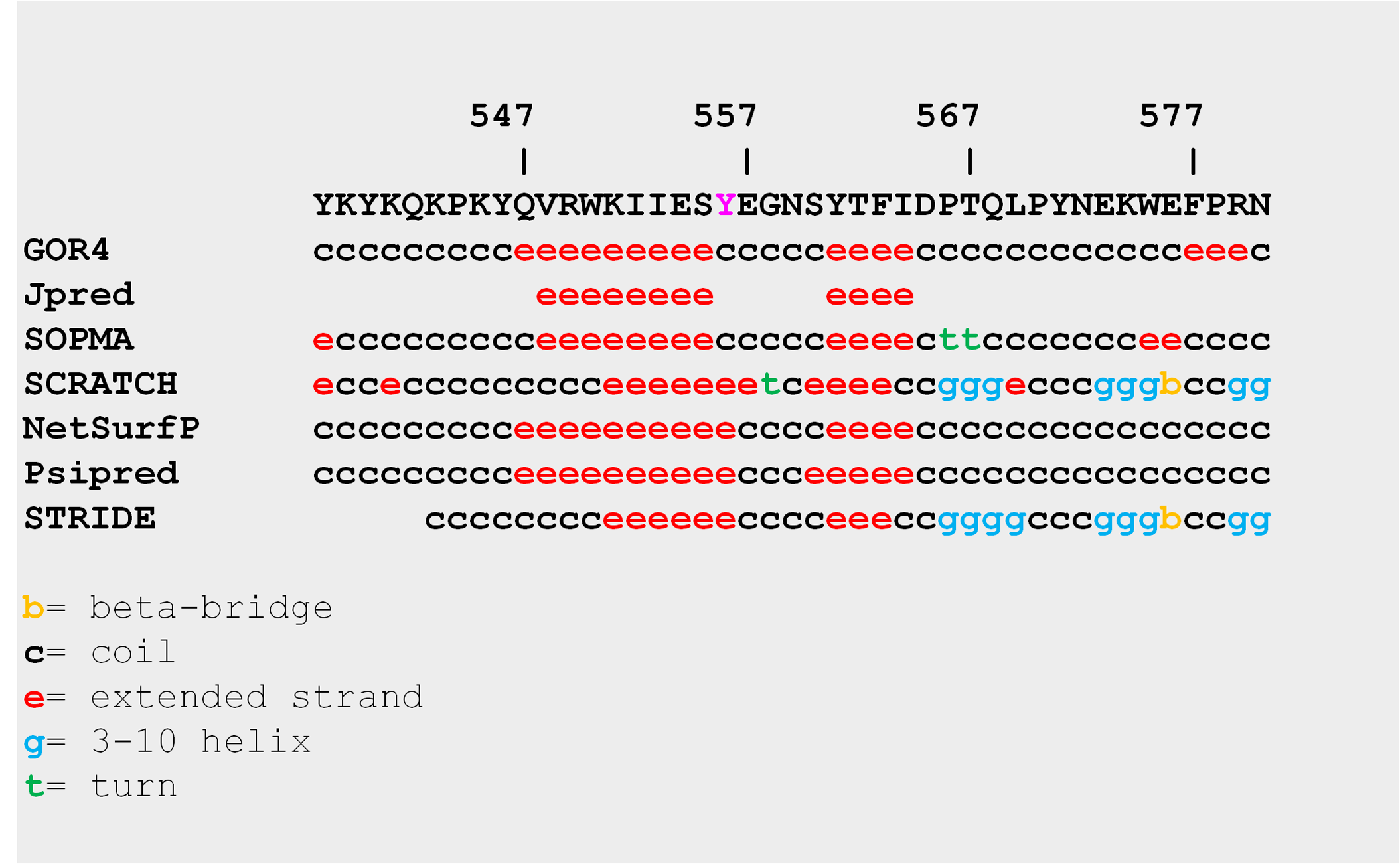

Supplement: Figure S2 — Secondary structure prediction of the JMR sequence (residues 538–580) from CSF-1RWT. Prediction was performed using sequence-based algorithms GOR4 [33], Jpred [34], SOPMA [42], SCRATCH [44], NetSurfP [45], Psipred [46] and a structure-based method STRIDE [47]. Predicted structural elements are coded as indicated at bottom. (TIF) [file pone.0097519.s002.tif]

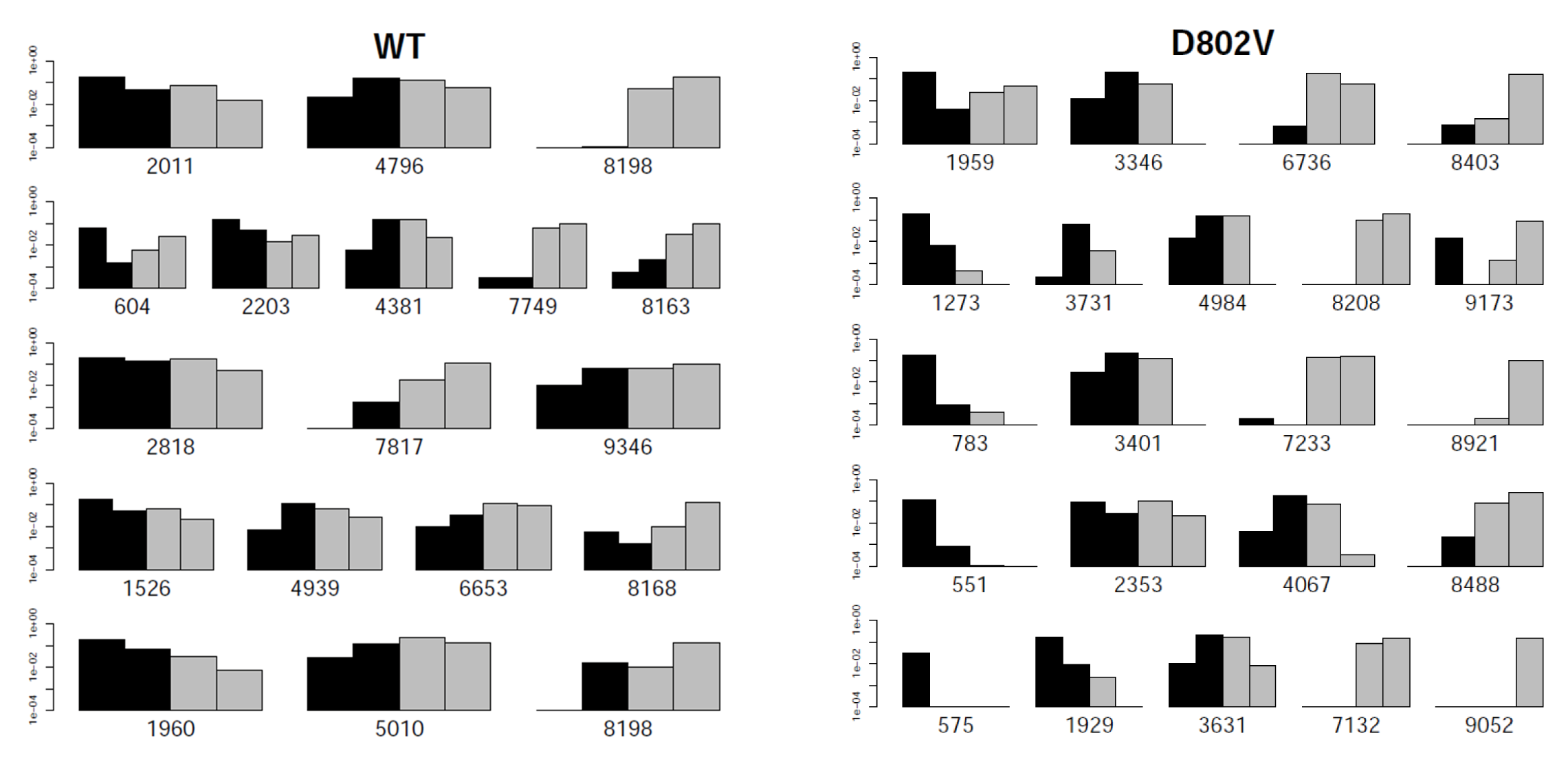

Supplement: Figure S3 — Convergence analysis of the MD simulations for CSF-1RWT (WT) and CSF-1RMU (D802V) models performed on the 90 ns concatenated trajectories. Grouping of MD conformations was made using five independent runs calculated for each model. The populations of each group for each run are presented as histograms in the logarithmic scale denoted by different colors, black and grey from the 1st and 2nd halves of the two replica respectively. The identification numbers of each reference structure denotes the time (ns) in which it was picked from the MD trajectory. The fourth run contains reference structures that are better represented in both replicas and it was chosen for further NM calculations. (TIF) [file pone.0097519.s003.tif]

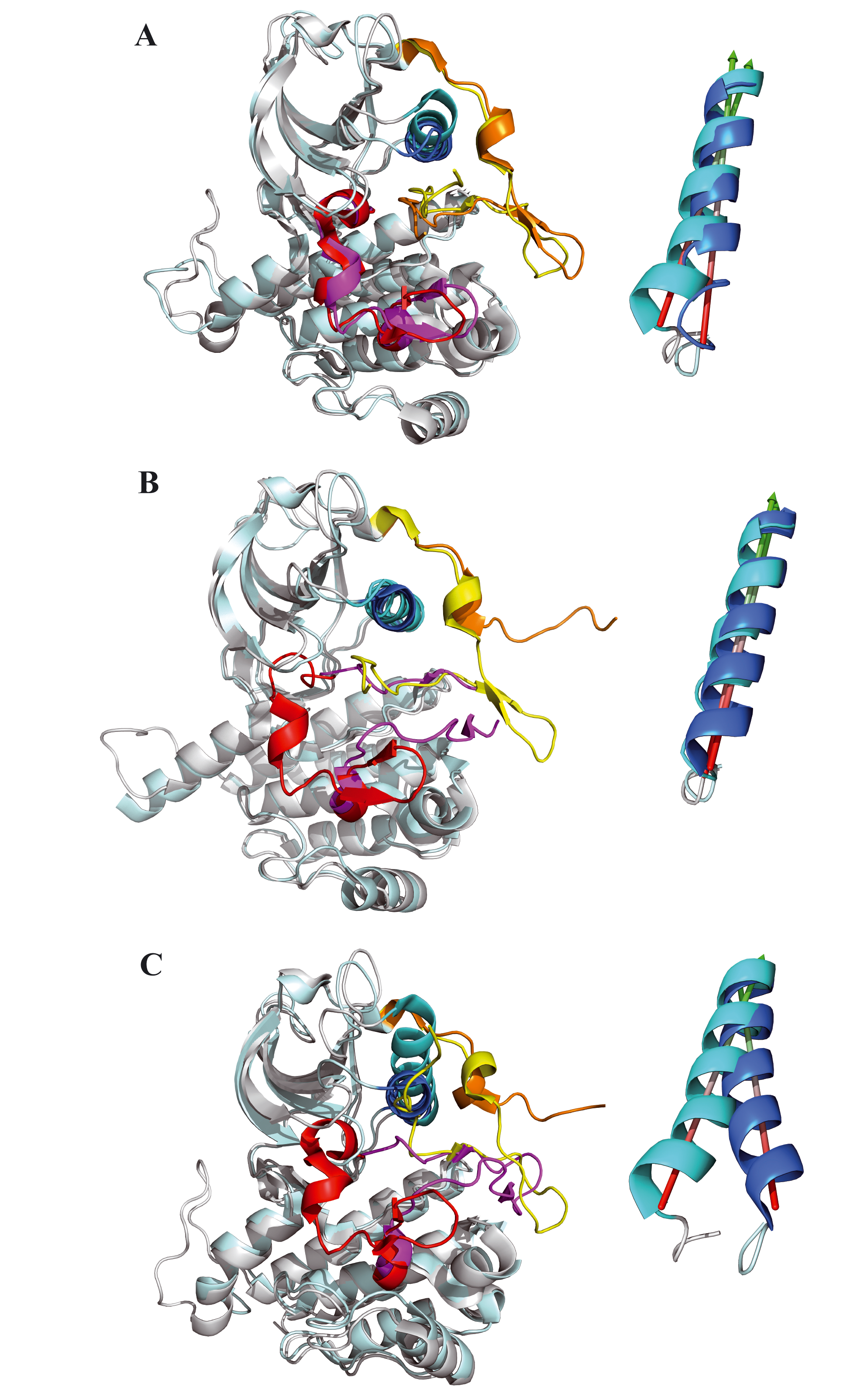

Supplement: Figure S4 — 3D structural mapping of the inter-residues communication in KITWT and KITMU. The average MD conformation is presented as cartoon. The proteins fragments are presented with different colors: JMR (blue), Cα-helix (violet), P-loop (yellow), C-loop (green) and A-loop (red). Communication pathways (CPs) between residues atoms (small circles) are depicted by coloured lines: CPs formed by the A-loop residues in orange; by the JMR-residues in magenta. The key residues in the communication networks are labelled (in KITWT) and depicted as bulky circles. (TIF) [file pone.0097519.s004.tif]

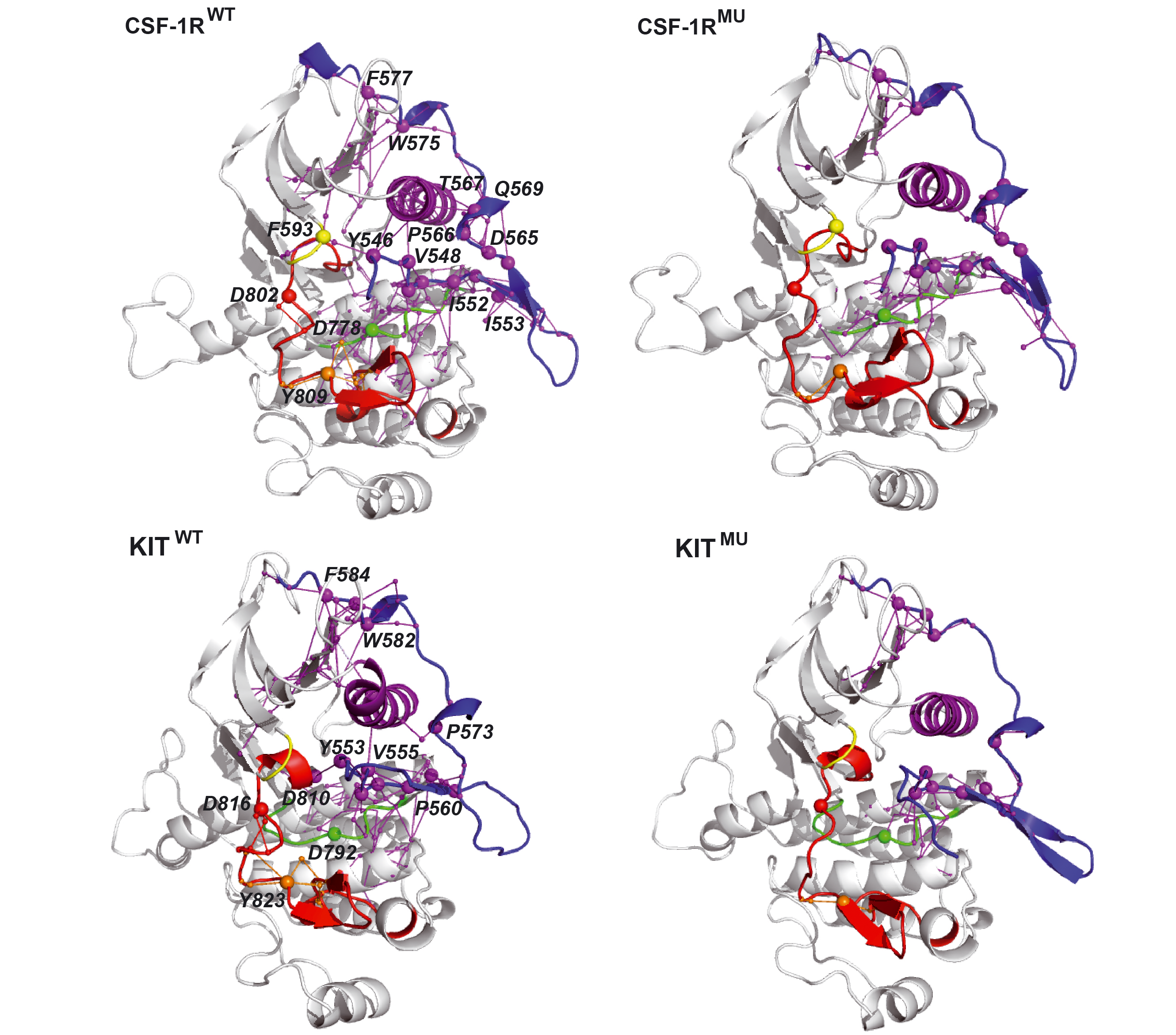

Supplement: Figure S5 — Structure of the cytoplasmic domain of CSF-1R and KIT in the native form. Superimposition of the CSF-1R and KIT crystallographic structures : (A) CSF-1R (2OGV [22]) and KIT (1T45 [21]) in the inactive conformation; (B) CSF-1R in the inactive (2OGV[22]) and the active conformations (3LCD [85]; (C) KIT in the inactive (1T45) and active (1PKG, [86]) conformations. The proteins are presented as cartoon, CSF-1R is in blue light and KIT is in grey light. The key structural fragments of receptors in the inactive and the active conformations are highlighted in color. The JMR is in yellow and in orange; the A-loop is in red and magenta; the Cα-helix is in cyan and blue. The relative orientation of the Cα-helix (inserts) in two proteins is presented together with the principal axis of helices detected with PyMol. (TIF) [file pone.0097519.s005.tif]
