# Supplementary material for: Differential Effects of CSF-1R D802V and KIT D816V Homologous Mutations on Receptor Tertiary Structure and Allosteric Communication
Source: PLoS One. 2014 May 14;9(5):e97519. doi: 10.1371/journal.pone.0097519 (PMC4020833; doi:10.1371/journal.pone.0097519)
Supplement: Table S1 — Characteristics of convergence analysis of the native CSF-1R (WT) and its mutant (D802V) MD trajectories. (DOC) [file pone.0097519.s006.doc]

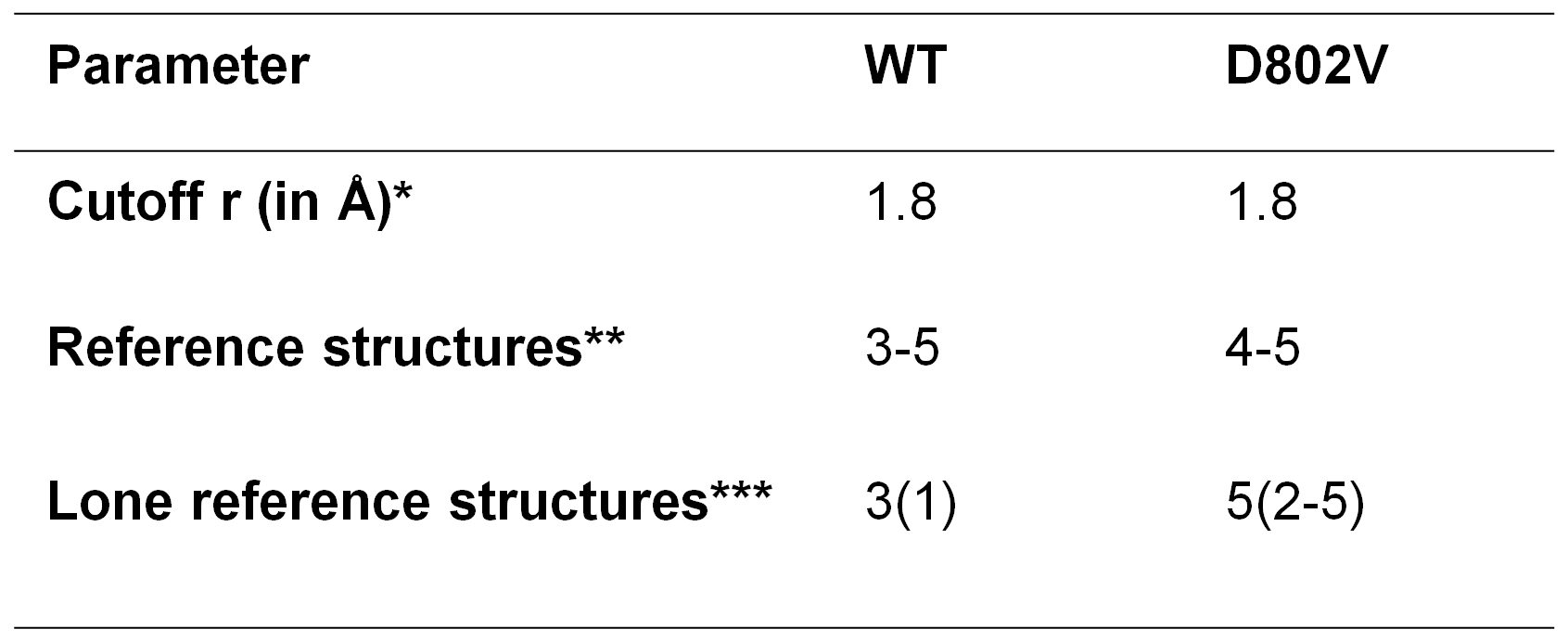


Notes:

* the best RMSDs cutoff to present the conformational diversity;

** the range of reference structures identified in the 5 runs;

*** the number of runs with lone reference structure together with the range of lone reference structures in the five runs.
